# The relationship between self-rated health and objective health status: a population-based study

**DOI:** 10.1186/1471-2458-13-320

**Published:** 2013-04-09

**Authors:** Shunquan Wu, Rui Wang, Yanfang Zhao, Xiuqiang Ma, Meijing Wu, Xiaoyan Yan, Jia He

**Affiliations:** 1 Department of Health Statistics, Second Military Medical University, No.800 of XiangYin Road, Shanghai, 200433, China

**Keywords:** Self-rated health, Health status, Disease prevalence, Laboratory parameters, Health-related factors

## Abstract

**Background:**

Self-rated health (SRH), a subjective assessment of health status, is extensively used in the public health field. However, whether SRH can reflect the objective health status is still debatable. We aim to reveal the relationship between SRH and objective health status in the general population.

**Methods:**

We assessed the relationship between SRH and objective health status by examining the prevalence of diseases, laboratory parameters, and some health-related factors in different SRH groups. Data were collected from 18,000 residents randomly sampled from the general population in five cities of China (3,600 in each city). SRH was assessed by a single-item health measure with five options: “very good,” “good,” “fair,” “bad,” and “very bad.” The differences in prevalence of diseases, laboratory parameters, and health-related factors between the “healthy” (very good plus good), “relatively healthy” (fair), and “unhealthy” (bad plus very bad) groups were examined. The odds ratios (ORs) referenced by the healthy group were calculated using logistic regression analysis.

**Results:**

The prevalence of all diseases was associated with poorer SRH. The tendency was more prominent in cardio-cerebral vascular diseases, visual impairment, and mental illnesses with larger ORs. Residents with abnormalities in laboratory parameters tended to have poorer SRH, with ORs ranging from 1.62 (for triglyceride) to 3.48 (for hemoglobin among men) in a comparison of the unhealthy and healthy groups. Most of the health-related factors regarded as risks were associated with poorer SRH. Among them, life and work pressure, poor spiritual status, and poor quality of interpersonal relationships were the most significant factors.

**Conclusions:**

SRH is consistent with objective health status and can serve as a global measure of health status in the general population.

## Background

Self-rated health (SRH) (also known as self-assessed health or self-perceived health) refers to a single-item health measure in which individuals rate the current status of their own health on a four- or five-point scale from *excellent* to *poor*. It is popular for its simplicity and has been extensively studied in Western populations. A series of national and international analyses has consistently shown that SRH is a good predictor of mortality of various diseases [1-3]. Furthermore, many researchers have attempted to investigate the factors related to SRH and have found that SRH is strongly associated with morbidity and disability [4-6]. Economic or social factors are also its main determinants [7-9].

SRH is a subjective reflection of health status, called “perceived” or “subjective” health. It has been widely studied in survey research [3,7,8]. However, most of the studies concerning SRH have focused on specific age groups, sex groups, or patient groups [10-12]. Studies reporting SRH among the general population are few. Relative to SRH, some indicators provide direct evidence to the health status of individuals, including previous and current diseases (diagnosed by physicians), and clinical parameters measured in the hospital; these have been termed as “actual” or “objective” health. Some health-related factors, such as demographic characteristics, health history, life habit, life stress and work strain, are closely associated with objective health.

There is a complex relationship between subjective and objective health [13]. Some studies reported that old people perceived their health in positive terms and tended to over-estimate their health [14-16], while other studies found that old people tended to report poorer health than others who were with similar objective health conditions [15,17,18]. Is subjective health consistent with objective health? Can SRH serve as a global measure of health status in the general population? The answers are still debatable. Thus, the assessment of the relationship between SRH and objective health status is important, for this determines whether SRH can serve as an indicator of objective health status.

## Methods

This study examined the association between SRH and objective health status through a comprehensive collection of data on disease prevalence, laboratory parameters, and health-related factors in a randomly selected sample from five cities of China: Shanghai, Beijing, Xi’an, Wuhan and Guangzhou. We attempted to determine the relationship between SRH and objective health status in this population sample and to identify the factors that best discriminate the different SRH groups.

### Study design and sample

We used a part of the data from our previous epidemiological survey on gastrointestinal diseases in five cities of China. The details of the methodology of the survey have been published elsewhere [19], and are briefly described here. The survey was administered to 18,000 residents aged 18–80 years (3,600 in each city) from April 2007 to January 2008. Eligibility criteria were based on age and the mental and physical ability of subjects to participate in the survey. Residents who were illiterate, not in the 18–80 age group, or suffering from psychiatric illnesses or other disabilities were excluded from the study. They were sampled using a randomized, stratified, multiple-stage sampling method, with the age/gender distribution of the sample in accord with the distribution of local population according to the population census statistics published by the government. Thus, the sample would not be affected by the original impetus of the survey. In the first stage, one or more districts from the urban stratum and one or more counties from the rural stratum were randomly selected from each region. In the second stage, one or more blocks from the urban districts and one or more townships from the rural counties were randomly selected. In the third stage, one or more residential areas from the urban blocks and one or more villages from the rural townships were randomly sampled. Questionnaires were self-completed, with trained interviewers giving explanation on any unclear questions. The entire procedure was overseen by the supervisors to ensure the quality of the survey.

### Measurements

Demographic information were gathered on gender, age, weight, height, marital status (married, unmarried, divorced, or widowed), educational level (less than primary school, completed only primary school, completed secondary school, completed high school, completed college/pre-university/university, master’s degree, or doctoral degree), total family monthly income (<RMB 2,000, RMB 2,000–4,999, RMB 5,000–9,999, or ≥RMB 10,000), etc. We used a single-item measure of subjective health—“In general, how would you rate your current health status?”—to assess self-ratings of health. Five options, which were recommended by the World Health Organization (WHO) [20] and the European Network for the Calculation of Health Expectancies (Euro-PEVES) 2 group [21], were listed: “very good,” “good,” “fair,” “bad,” or “very bad.”

To assess the relationship between SRH and objective health status, subjects were asked to report whether they had been diagnosed with chronic diseases, including hypertension, cerebrovascular disorder, diabetes mellitus, and chronic bronchitis. Blood sampling was carried out after overnight fasting at the Second Military Medical University Changhai Hospital in Shanghai, but this was not done in the other four cities. The following parameters were measured to assess the subjects’ health status: red blood cell (RBC), hemoglobin (HGB), aspartate aminotransferase (AST), total cholesterol (TC), triglyceride (TG), low-density lipoprotein cholesterol (LDL-C), and fasting plasma glucose (FPG). RBC and HGB indicate the body’s ability to transport oxygen to tissues. AST and TC are indicators that mainly reflect hepatic dysfunction. High levels of TG and LDL-C are two major risk factors for cardiovascular and cerebral diseases. Plasma glucose provides the energy cells require and must be maintained at a certain level in order to sustain the body’s needs. Body mass index (BMI) was calculated as weight in kilograms divided by the square of height in meters, and the WHO criterion for Asian populations was used to classify subjects as obese, overweight, normal weight, and underweight [22]. Data on some health-related factors were also collected, including body weight five years ago, tobacco use, frequency of physical activity, life stress, work strain, spiritual status, and quality of interpersonal relationships. Tobacco use was measured with a single question: “Do you currently smoke?” Six options were given: “no,” “1–5 cigarettes/day,” “6–10 cigarettes/day,” “11–15 cigarettes/day,” “16–20 cigarettes/day,” or “≥21 cigarettes/day.” Frequency of physical activity was measured with the following question: “How often do you engage in physical activities (e.g., physically active work, walking, riding a bike, and doing exercises)?” Four options were given: “never,” “<4 times per month,” “at least once a week,” or “at least once a day.” Life stress was measured by asking “Overall, do you feel stress in your daily life?” and work strain was measured by asking “Overall, do you feel stress in your daily work?” Life stress and work strain were assessed on a five-point scale: “no,” “a little,” “moderate,” “quite a lot,” and “extreme.” Spiritual status was measured by asking “Overall, how would you rate your spirituality?” and quality of interpersonal relationships was measured by asking “Overall, how would you rate your quality of interpersonal relationships?” Spiritual status and quality of interpersonal relationships were assessed by “very good,” “good,” “moderate,” “bad,” or “very bad.” Women were also asked to answer their ages at menopause.

### Statistical analysis

All the data were doubly input into the database by two independent professional data processors using EpiData 3.1. Statistical Analysis System (SAS) 9.1.3 was used for analyzing the survey data. Data are expressed as mean±standard deviation (SD). For statistical comparison, the differences among the “healthy” (very good plus good), “relatively healthy” (fair), and “unhealthy” (bad plus very bad) groups were examined, because it was difficult to determine whether “fair” should be defined as “good health” or “poor health,” and because only a few subjects rated their health as very good (1,770 subjects) or very bad (58 subjects). Statistical significance for laboratory parameters among different groups was determined by using an analysis of variance with Bonferroni’s multiple-comparison tests. The odds ratios (ORs) and 95% confidence intervals (CIs) for the “relatively healthy” and “unhealthy” groups were calculated referenced by the “healthy” group, using logistic regression analysis by controlling for gender, age, region, marital status, educational level, current work, family income, current smoking, current drinking, frequency of physical activity, and chronic diseases. All hypothesis tests used two-sided tests and a p-value less than 0.05 was considered statistically significant.

### Ethics

All the subjects had been informed that their records might be used for analysis, and had signed a written informed consent form before participation. The present study was approved by the ethics committee of Second Military Medical University.

## Results

### Demographic information

Among the 18,000 subjects, 16,091 actually answered the questionnaires; however, 13 were excluded from the analysis because of considerable missing data or logistic errors, and another 4 were excluded because they did not answer the general health item. Data from a total of 16,074 subjects were utilized in the statistical analysis, including 8,388 females (52.18%) and 7,686 males (47.82%). These subjects had very little missing data (less than 1%), and we excluded the subjects from relevant analysis if they do have missing data on certain items. Subjects’ demographic details are shown in Table 1. The mean age was 42.6 (±15.2) years. Among them, 1,770 subjects (11.01%) rated their health as very good, 7,581 (47.16%) as good, 5,803 (36.10%) as fair, 862 (5.36%) as bad, and 58 (0.36%) as very bad. Age was negatively correlated with health rating. A total of 3,151 subjects completed the blood sampling in Shanghai with data suitable for analysis, including 1,749 females (55.51%) and 1,402 males (44.49%), with mean age of 47.7 (±14.1) years. A total of 164 subjects (5.20%) rated their health as very good, 1,347 (42.75%) as good, 1,492 (47.35%) as fair, 141 (4.47%) as bad, and 7 (0.22%) as very bad. Similarly, the older the subjects were, the poorer they rated their health. The objective health status of the “healthy” (very good plus good), “relatively healthy” (fair), and “unhealthy” (bad plus very bad) groups were compared.

**Table 1:** Demographic information of the subjects

	**n (female/male)**	**Mean age (SD)**	p- value*
Subjects in all five cities			
Total	16074 (8388/7686)	42.6 (15.2)	
SRH			
very good	1770 (845/925)	34.7 (12.7)	<0.001
good	7581 (3773/3808)	40.1 (14.3)	
fair	5803 (3233/2570)	46.0 (15.0)	
bad	862 (506/356)	55.0 (14.6)	
very bad	58 (31/27)	53.8 (16.2)	
Subjects in Shanghai who have completed the blood sampling			
Total	3151 (1749/1402)	47.7 (14.1)	
SRH			
very good	164 (74/90)	41.5 (13.6)	<0.001
good	1347 (694/653)	44.6 (14.0)	
fair	1492 (890/602)	50.3 (13.2)	
bad	141 (88/53)	58.0 (13.0)	
very bad	7 (3/4)	47.6 (16.5)	

* p-values for mean ages.

### Disease prevalence

Table 2 presents the prevalence of various diseases by SRH status, including cardio-cerebral vascular diseases, digestive diseases, respiratory diseases, and mental illnesses. Logistic regression analysis showed that significant increases in the prevalence of all diseases were associated with a lower SRH, adjusting for gender, age, region, marital status, educational level, current work, family income, current smoking, current drinking, frequency of physical activity, and other chronic diseases. The ORs were at least twofold when comparing the unhealthy group with the healthy group, apart from for chronic pharyngitis (OR 1.65 [1.29, 2.11]) and osteoarthritis (OR 1.54 [1.13, 2.09]), and there were larger ORs for cardio-cerebral vascular diseases, visual impairment, and mental illnesses. As for tristimania, the OR was 14.14 [5.60, 35.73].

**Table 2:** Disease prevalence by self-rated health status

	**Healthy**	**Relatively healthy**		**Unhealthy**	
	***n *(%)**	***n *(%)**	**OR [95% CI]**	***n *(%)**	**OR [95% CI]**
Hypertension	615 (6.58)	1017 (17.53)	1.83 [1.62, 2.06]	282 (30.65)	2.61 [2.16, 3.15]
Cerebrovascular disorder	93(0.99)	222 (3.83)	2.00 [1.54, 2.58]	119 (12.93)	4.82 [3.53, 6.58]
Angina	53 (0.57)	122 (2.10)	1.88 [1.34, 2.63]	80 (8.70)	6.00 [4.07, 8.86]
Diabetes mellitus	85 (0.91)	238 (4.10)	2.86 [2.20, 3.72]	90 (9.78)	6.36 [4.54, 8.92]
Dyspepsia	351 (3.75)	525 (9.05)	1.81 [1.56, 2.10]	137 (14.89)	2.76 [2.19, 3.49]
Gastritis	693 (7.41)	966 (16.65)	1.74 [1.56, 1.95]	213 (23.15)	2.37 [1.96, 2.87]
Peptic ulcer	213 (2.28)	323 (5.57)	1.63 [1.35, 1.97]	86 (9.35)	2.50 [1.88, 3.33]
IBD	100 (1.07)	146 (2.52)	1.75 [1.33, 2.28]	51 (5.54)	3.14 [2.16, 4.58]
Liver disorder	196 (2.10)	324 (5.58)	2.00 [1.65, 2.43]	80 (8.70)	3.58 [2.65, 4.83]
Gallbladder disorder	241 (2.58)	353 (6.08)	1.43 [1.19, 1.70]	94 (10.22)	2.13 [1.62, 2.80]
Renal disorder	120 (1.28)	212 (3.65)	2.23 [1.76, 2.84]	84 (9.13)	4.41 [3.20, 6.07]
Chronic bronchitis	183 (1.96)	346 (5.96)	2.05 [1.69, 2.48]	100 (10.87)	2.34 [1.77, 3.10]
Asthma	45 (0.48)	75 (1.29)	1.57 [1.06, 2.32]	35 (3.80)	3.57 [2.18, 5.86]
Chronic pharyngitis	518 (5.54)	654 (11.27)	1.65 [1.45, 1.88]	100 (10.87)	1.65 [1.29, 2.11]
Chronic cough	98 (1.05)	188 (3.24)	1.90 [1.46, 2.45]	55 (5.98)	2.45 [1.70, 3.53]
Eczema	80 (0.86)	136 (2.34)	1.98 [1.48, 2.66]	28 (3.04)	3.29 [2.04, 5.30]
Rheumatoid arthritis	267 (2.86)	352 (6.07)	1.44 [1.21, 1.71]	169 (18.37)	2.48 [1.96, 3.13]
Osteoarthritis	176 (1.88)	289 (4.98)	1.61 [1.31, 1.97]	73 (7.93)	1.54 [1.13, 2.09]
Severe visual impairment	35 (0.37)	60 (1.03)	1.69 [1.09, 2.62]	35 (3.80)	4.58 [2.70, 7.78]
Anxiety neurosis	15 (0.16)	28 (0.48)	1.98 [1.07, 2.90]	14 (1.52)	4.48 [2.05, 9.76]
Tristimania	8 (0.09)	32 (0.55)	4.67 [2.15, 10.13]	15 (1.63)	14.14 [5.60, 35.73]

n=16 074.

The odds ratios were calculated referenced by the “healthy” group, using logistic regression analysis by controlling for gender, age, region, marital status, educational level, current work, family income, current smoking, current drinking, frequency of physical activity, and other chronic diseases.

IBD: inflammatory bowel disease.

### Laboratory parameters

Laboratory parameters of the three groups by SRH status are presented in Table 3. RBC and HGB were significantly lower in the relatively healthy and unhealthy groups when compared with the healthy group. Levels of AST, TC, TG, LDL-C, and FPG were significantly higher in the relatively healthy and unhealthy groups than in the healthy group.

**Table 3:** Subjects’ laboratory parameters by self-rated health status

	**Healthy**	**Relatively healthy**	**Unhealthy**
RBC (10^12^/L)	4.76±0.48	4.68±0.45^*^	4.59±0.45^*^
HGB (g/L)	138.8±16.1	136.7±15.9^*^	135.1±15.5^*^
AST (U/L)	23.4±11.1	24.5±13.2^*^	26.0±11.9^*^
TC (mmol/L)	4.85±1.46	5.00±0.96^*^	5.21±1.17^*^
TG (mmol/L)	1.40±1.15	1.52±1.21^*^	1.61±1.18^*^
LDL-C (mmol/L)	3.12±0.83	3.22±0.84^*^	3.24±0.82^*^
FPG (mmol/L)	5.17±1.21	5.38±1.55^*^	5.58±1.66^*^

n=3151.

Data are mean ± SD.

Statistical significance was determined using Bonferroni’s multiple-comparison tests.

* p<0.05, comparing with the healthy group.

RBC: red blood cell.

HGB: hemoglobin.

AST: aspartate aminotransferase.

TC: total cholesterol.

TG: triglyceride.

LDL-C: low-density lipoprotein cholesterol.

FPG: fasting plasma glucose.

Abnormalities in laboratory parameters by SRH status adjusted for gender (except for RBC and HGB), age, marital status, educational level, current work, family income, current smoking, current drinking, and frequency of physical activity are shown in Table 4. The cut-off values for abnormalities were defined by Changhai Hospital according to the criteria for the Chinese people. For RBC, HGB among women, TC, and FPG, significantly higher ORs were observed in the relatively healthy and unhealthy groups than in the healthy group. For HGB among men, AST, TG, and LDL-C, the ORs were significantly higher in the unhealthy group than in the healthy group.

**Table 4:** Laboratory parameters abnormalities by self-rated health status

**Cut-off values for abnormalities**	**Healthy**	**Relatively healthy**		**Unhealthy**	
	***n *(%)**	***n *(%)**	**OR [95% CI]**	***n *(%)**	**OR [95% CI]**
RBC < 3.5×10^12^/L^a^	54 (7.03)	79 (8.88)	1.58 [1.09, 2.17]	10 (10.99)	1.96 [1.04, 3.89]
RBC < 4.0×10^12^/L^b^	11 (1.48)	23 (3.82)	2.98 [1.54, 5.72]	3 (5.26)	3.24 [1.47, 10.24]
HGB < 110 g/L^a^	35 (4.56)	58 (6.52)	1.43 [1.02, 2.05]	8 (8.79)	1.92 [1.15, 4.20]
HGB < 120 g/L^b^	16 (2.15)	18 (2.99)	1.38 [0.74, 2.94]	4 (7.02)	3.48 [1.23, 6.04]
AST >= 64 U/L	14 (0.93)	28 (1.88)	1.64 [0.85, 3.12]	4 (2.70)	2.70 [1.65, 4.19]
TC > 5.8 mmol/L	102 (6.75)	158 (10.59)	1.30 [1.01, 1.70]	24 (16.22)	1.72 [1.05, 2.84]
TG > 2.0 mmol/L	244 (16.15)	293 (19.64)	1.17 [0.96, 1.43]	37 (25.00)	1.62 [1.07, 2.47]
LDL-C > 4.9 mmol/L	40 (2.65)	43 (2.88)	0.86 [0.55, 1.36]	12 (8.11)	2.31 [1.13, 4.69]
FPG > 6.2 mmol/L	119 (7.88)	194 (13.00)	1.49 [1.15, 1.91]	26 (17.57)	1.84 [1.09, 2.93]

n=3151.

The odds ratios were calculated referenced by the “healthy” group, using logistic regression analysis by controlling for gender (except for RBC and HGB), age, marital status, educational level, current work, family income, current smoking, current drinking, frequency of physical activity.

^a^ calculated only among women.

^b^ calculated only among men.

For abbreviations see Table 3.

### Health-related factors

The health-related factors regarded as risks are shown in Table 5. For underweight, marital status, low income, physical activities, life stress, work strain, spiritual status, and quality of interpersonal relationships, significantly higher ORs were observed in the relatively healthy and unhealthy groups than in the healthy group. The ORs for weight loss, low level of education, and early menopause were significantly higher in the unhealthy group than in the healthy group. Among all the factors, life and work pressure, poor spiritual status, and bad quality of interpersonal relationships had larger ORs, which indicates that these factors had a considerable impact on people’s health. The ORs for obesity and smoking were not statistically significant. All the ORs were adjusted by gender (except for “age at menopause”), age, region, health-related factors (including marital status, educational level, current work, family income, current smoking, current drinking, frequency of physical activity, but excluding itself), and chronic diseases, using logistic regression analysis.

**Table 5:** Health-related factors regarded as risks by self-rated health status

	**Healthy**	**Relatively healthy**		**Unhealthy**	
	***n *(%)**	***n *(%)**	**OR [95% CI]**	***n *(%)**	**OR [95% CI]**
BMI: < 18.5 kg/m^2^	905 (9.68)	536 (9.24)	1.26 [1.11, 1.42]	110 (11.96)	1.86 [1.47, 2.36]
Weight loss: ≥5 kg, compared with 5 years ago	502 (5.37)	440 (7.58)	1.14 [0.99, 1.32]	150 (16.30)	2.01 [1.62, 2.50]
Marital status: divorced/widowed	249 (2.66)	340 (5.86)	1.37 [1.14, 1.65]	122 (13.26)	1.69 [1.30, 2.21]
The highest level of education: less than primary school	593 (6.34)	612 (10.55)	0.98 [0.85, 1.14]	282 (30.65)	1.59 [1.29, 1.97]
Total income of family per month: <2000 Yuan	4942 (52.85)	3190 (54.97)	1.09 [1.01, 1.18]	681 (74.02)	1.59 [1.33, 1.90]
Current smoking: >20 cigarettes per day	382 (4.09)	248 (4.27)	1.02 [0.85, 1.23]	50 (5.43)	1.16 [0.83, 1.63]
Age of menopause: ^a^ ≤45 years old	196 (4.24)	238 (7.36)	1.09 [0.88, 1.34]	105 (19.55)	1.68 [1.26, 2.25]
Frequency of physical activity: < 4 times per month	1827 (19.54)	1421 (24.49)	1.56 [1.44, 1.70]	246 (26.74)	2.39 [2.02, 2.84]
Life stress: quite a lot/extreme	686 (7.34)	837 (14.42)	2.33 [2.08, 2.62]	292 (31.74)	6.98 [5.79, 8.42]
Work strain: quite a lot/extreme	560 (5.99)	590 (10.17)	2.07 [1.81, 2.35]	187 (20.33)	5.32 [4.31, 6.57]
Spiritual status: bad/very bad	45 (0.48)	204 (3.52)	6.92 [4.96, 9.66]	282 (30.65)	66.03 [46.37, 94.02]
Quality of interpersonal relationships: bad/very bad	68 (0.73)	129 (2.22)	2.54 [1.86, 3.46]	102 (11.09)	10.24 [7.14, 14.69]

n=16 074.

The odds ratios were calculated referenced by the “healthy” group, using logistic regression analysis by controlling for gender (except for “age of menopause”), age, region, health-related factors (including marital status, educational level, current work, family income, current smoking, current drinking, frequency of physical activity, but excluding itself), and chronic diseases.

^a^ calculated only among women.

## Discussion

This study aimed to determine whether SRH could reflect objective health status and serve as a global measure of health status in the general population. For this purpose, we examined the relationships between SRH and the following: (1) prevalence of diseases diagnosed by the physicians, (2) laboratory parameters examined in the hospital, and (3) health-related factors that were regarded as risks. We found that lower SRH was associated with significant increases in the prevalence of all the diseases and abnormalities in laboratory parameters. Some health-related factors, including underweight, weight loss, marital status, education, low income, early menopause, physical activities, life stress, work strain, spiritual status, and quality of interpersonal relationships, were significantly associated with SRH. This suggests that individuals may use these factors to evaluate their overall health [23,24].

Notably, most subjects rated their health positively, for only 5.72% subjects reported bad or very bad health, whereas nearly 60% rated their health as very good or good. A survey conducted in Sweden among subjects aged 18–79 years showed that 7% of the men and 9% of the women had poor SRH [25]; thus, the prevalence rate of poor SRH in our population sample, with a similar age range as theirs, was slightly lower. An obvious higher mean age was observed as the subjects rated poorer health. This means that the subjective health status of the population significantly decreases with advancing age. Similar results were found in previous studies, which showed that age was a risk factor for poor SRH [25-29].

The prevalence of all the diseases included in our study, which was an important indicator of objective health, contributed to decreased SRH. This means that SRH has the ability to distinguish patients from relatively healthy people. It is noteworthy that larger ORs were found for cardio-cerebral vascular diseases, visual impairment, and mental illnesses when comparing the unhealthy group with the healthy group. An earlier study reported that with China’s rapid economic development, diseases of the heart and the cerebrovascular system have become two leading causes of deaths in this country, ranking second and third among men, and first and second among women [30]. It is understandable that patients with cardio-cerebral vascular diseases are more likely to worry about their future, and that the diseases cause great cost and burden to themselves and their families; thus, people who suffer from these diseases rated poorer health. Visual impairment is associated with a decreased ability to perform activities of daily living and poor quality of life, particularly in the sense of depression and reduced social interaction [31], and patients with mental illnesses had more impairment in quality of life than those with common medical disorders [32]. These could explain why patients with visual impairment or mental illnesses rated poorer health. A previous study conducted in Sweden showed that many chronic diseases, such as neurological disease, rheumatoid arthritis, and cancer, are strongly associated with poor SRH [29]. This conclusion has been further strengthened with the evidence from our study.

Studies examining the association of SRH with laboratory parameters were still limited. In order to further elucidate this relationship in our study, we examined the laboratory parameters in the subjects who had experienced blood sampling and listed the laboratory parameters with statistical significances. We found that better SRH was associated with increased RBC and HGB, and decreased AST, TC, TG, LDL-C, and FPG. Furthermore, subjects with abnormal laboratory parameters reported poorer health. Laboratory parameters can objectively reflect health status and are of fundamental importance for the diagnosis, prognosis and treatment of various diseases [33]. The consistency of SRH and objective health status assessed by laboratory parameters is further evidence that SRH is a good indicator of health status.

In addition, the relationship between SRH and some health-related factors, which were regarded as risk factors for health, were examined. Obesity is surprisingly unrelated to poorer SRH, and this finding is inconsistent with many previous studies [24,25,34,35]. On further consideration, this is also explicable. In Chinese culture, being fat is not a signal of unhealthiness but a symbol of acquiring good fortune, because only the more wealthy people can afford to eat more and can thus put on more weight [36]. This is quite different in Western countries. Underweight and weight loss are two risk factors observed in this study. Underweight or weight loss was linked to eating disorders such as anorexia nervosa, or consumptive diseases such as malignant tumors. The findings also support marriage as a protective factor for health [37]: subjects who were divorced or widowed rated poorer health. Subjects with higher income tended to be more optimistic about their health, because they have been observed to practice more health promoting behaviors such as good dietary intake practices and physical activity [38]. Shibuya et al. also found that low income was associated with poor SRH in a Japanese sample [7]. Lim et al. found that marital status, education, household income, current smoking, and exercise were associated with SRH in a Singaporean sample [39]. Life stress and work strain as risk factors for poor SRH have been examined less frequently in population studies. One study has demonstrated that family stress is a risk factor for unfavorable health-related outcomes [40], and another has revealed that work stress is associated with poor SRH [41]. These results support the conclusion of this study.

Our results need to be interpreted within the context of the study limitations. First, the cross-sectional nature of the study did not allow us to investigate the temporal mechanisms between SRH on the one hand and diseases or abnormalities in laboratory parameters or risk factors on the other. Second, we relied on a self-administrated questionnaire for measuring the clinical and health-related factors that were used to validate SRH. Except for laboratory parameters, all data relied on self-report. Like any other questionnaire study, the subjects’ answers may be affected by various kinds of bias such as social desirability. Finally, the study was a part of a larger study; therefore, the research questions can only be pursued within the boundaries of the original study. Some indicators were not integrated in our study due to the original focus of the study. Nevertheless, information on the majority of, but not all, possible confounders was available for analysis. There were also other factors associated with SRH such as depression, psychological well-being, health service utilization, medication usage, cognitive capacity, and social networks, but data on these factors were not collected in our study. However, with the information collected, we were able to explain the relationship between SRH and the objective health status.

The strengths of this study are as follows: (1) it includes a random sample of population from five cities of China with a broad range of ages in accord with the distribution of local population, which is a better representation of the total population. In contrast the majority of previous studies focusing on SRH mainly examined older population samples [5,13-15,17,18,42,43] or subjects with certain specific diseases [23,44-46]. (2) The present study investigates the association between SRH and objective health status from multiple dimensions, using a wide range of variables, including the prevalence of diseases, abnormalities in laboratory parameters, and health-related factors.

## Conclusion

This study provides evidence of the association between SRH and objective health status exemplified by the prevalence of diseases, abnormalities in laboratory parameters, and health-related factors. The results lead to a conclusion that SRH can reflect the objective health status and serve as a global measure of health status in the general population. This is very important since there is hardly any individual indicator that can comprehensively evaluate a person’s health status. Most of the factors influencing the SRH can be changed so as to improve health, for example, by actively treating diseases, engaging in more physical activity in daily life, improving mental well-being, and promoting adoption of healthy behaviors. It is therefore imperative for the public health sector to implement health promotion and treatment programs to target these specific goals in the community. Additionally, SRH could be used as a population-screening tool in the Chinese health care system, which could help public health officials identify those who are most in need of their services. Further studies to qualitatively and quantitatively explore these and other related variables might further advance our understanding of SRH and provide insights into the influencing factors of SRH.

## Abbreviations

SRH: Self-rated health; ORs: Odds ratios; WHO: World Health Organization; Euro-REVES: European Network for the Calculation of Health Expectancies; RBC: Red blood cell; HGB: Hemoglobin; AST: Aspartate aminotransferase; TC: Total cholesterol; TG: Triglyceride; LDL-C: Low-density lipoprotein cholesterol; FPG: Fasting plasma glucose; BMI: Body Mass Index; SAS: Statistical Analysis System; SD: Standard deviation; CIs: Confidence intervals.

## Competing interests

SQW, RW, YFZ, XQM, MJW, and XYY have no conflict of interests. JH has served as the director of the Department of Health Statistics, Second Military Medical University.

## Authors’ contributions

JH conceived of the study and supervised all aspects of its implementation. SQW and RW assisted with the survey, completed the statistical analyses and led the writing of different versions of the manuscript. YFZ, MJW and XYY assisted with the study, and XQM assisted with the survey and data analyses. All authors contributed to conceptualize ideas, interpret findings, and review the drafts of the manuscript, and they approved the final manuscript.

## Pre-publication history

The pre-publication history for this paper can be accessed here:

http://www.biomedcentral.com/1471-2458/13/320/prepub
